# 肺癌合并肺栓塞危险因素及预后的临床分析

**DOI:** 10.3779/j.issn.1009-3419.2011.10.03

**Published:** 2011-10-20

**Authors:** 峻 王, 卫华 周, 林 许, 民 杨, 丽娟 孟, 卫飞 樊, 骁麟 蒲, 媛华 杨

**Affiliations:** 1 210024 南京，江苏省老年研究所肿瘤内科 Department of Chemotherapy, Geriatric Institute of Jiangsu Province, Nanjing 210024, China; 2 210024 南京，江苏省老年研究所急诊科 2Department of Emergency, Geriatric Institute of Jiangsu Province, Nanjing 210024, China; 3 100043 北京，北京朝阳医院呼吸科 Department of Thoracic Surgery, Affiliated Cancer Hospital of Nanjing Medical University, Nanjing 210000, China; 4 210000 南京，南京医科大学附属肿瘤医院胸外科 Department of Respiratory Medicine, Beijing ChaoYang Hospital, Beijing 100043, China

**Keywords:** 肺肿瘤, 肺栓塞, 危险因素, 预后, Lung neoplasms, Pulmonary thromboembolism, Risk factor, Prognosis

## Abstract

**背景与目的:**

已有的研究结果表明恶性肿瘤常合并静脉血栓形成和肺栓塞，并以肺癌最常见。但是肺癌合并肺栓塞的发生机制和危险因素尚未完全明了。本研究的目的是总结我院54例肺癌合并肺栓塞的临床资料，并探讨其危险因素及预后。

**方法:**

对1999年4月-2010年1月江苏省肿瘤医院和江苏省老年研究所收治的肺癌合并肺栓塞患者进行回顾性病例-对照研究。单因素分析后，对可能有意义的因素再行条件*logistic*回归分析，寻找肺栓塞相关危险因素，同时应用*Log*-*rank*检验分析伴有和不伴有肺栓塞肺癌患者的生存差异。

**结果:**

本研究纳入54例肺癌合并肺栓塞患者和162例不伴有肺栓塞肺癌作对照。单因素分析时选择*P* < 0.20的变量进入回归模型进一步分析。回归模型结果发现接受化疗治疗的患者发生肺栓塞风险的OR值为2.64，Ⅲ期-Ⅳ期肺癌合并肺栓塞的OR值为2.25，腺癌合并肺栓塞的OR值为2.12，血红蛋白>140 g/L的肺癌患者合并肺栓塞的OR值为2.10，均具有统计学差异（*P* < 0.05）。合并肺栓塞的肺癌患者生存时间明显低于不合并肺栓塞的肺癌患者（*P*=0.02）。

**结论:**

化疗、晚期肿瘤、肺腺癌和高血红蛋白是肺癌合并肺栓塞的重要危险因素；合并肺栓塞的肺癌患者的生存时间明显低于无肺栓塞的肺癌患者。

## Abstract (en)

**Background and objective:**

Malignant tumors often combined with venous thrombosis and pulmonary embolism, especially in lung cancer. It has been proven that, the mechanisms and risk factors for lung cancer patients contracting pulmonary embolism are unclear. The aim of this study is to summarize the clinical data on 54 patients with lung cancer and concomitant pulmonary embolism, and to analyze the risk factors and prognosis of lung cancer with pulmonary thromboembolism (PTE).

**Methods:**

From April 1999 to January 2010, the clinical presentation of lung cancer patients with PTE from the Jiangsu Cancer Hospital and the Jiangsu Gerontic Institute were evaluated in the present retrospective study. Univariate analysis was conducted to determine the possible associated variables. Conditional *logistic* regression analysis was applied to explore risk factors of pulmonary embolism. Patient survival was also compared with matched controls via a *Log*-*rank* test.

**Results:**

A total of 54 lung cancer patients with PTE, matched with 162 lung cancer patients as controls, were included. In the univariate analysis, a *P* < 0.20 was considered as possible risk factor, which was included into the *logistic* regression model. The *logistic* regression model showed that the OR combined with pulmonary embolism was 2.64 in patients receiving chemotherapy, 2.25 in patients with stage Ⅲ-Ⅳ disease, 2.39 in patients combined with chronic obstructive pulmonary disease (COPD), 2.12 in patients with adenocarcinoma, 2.10 in patients with serum hemoglobin>140 g/L, and 1.76 in patients with central venous catheters. A significant difference was observed among the groups that received chemotherapy, adenocarcinoma, stage Ⅲ-Ⅳ disease and high henoglobin (*P* < 0.05). The survival time in patients with pulmonary embolism was remarkably lower than that in patients without pulmonary embolism (*P*=0.02).

**Conclusion:**

Chemotherapy, late stage disease and high serum hemoglobin are important risk factors for lung cancer patients with concomitant pulmonary embolism. The survival time of these patients is significantly lower than that in patients without pulmonary embolism.

早在1868年，Trousseau就注意到了肿瘤和静脉血栓形成的联系。有研究^[1, 2]^报道深静脉血栓形成（deep vein thrombosis, DVT）或肺血栓栓塞（pulmonary thromboembolism, PTE）在肿瘤患者中发生率高达4%-20%，且发生率依肿瘤类型和分期而异，其中肺癌为报道最常见发生血栓栓塞的恶性肿瘤之一。2005年，Blom等^[3]^发现恶性肿瘤患者发生血栓症的风险为普通人群的7倍。2009年，Chuang等^[4]^报道除了肿瘤本身以外，化疗、手术、静脉留置管、制动、基础病、吸烟等均为增加肺栓塞发生的危险因素。尽管静脉血栓栓塞症（venous thromboembolism, VTE）包括DVT和PTE，但两者有着较大的差异，其中最主要的区别是预后的明显差异。DVT常导致静脉炎后综合症，然而PTE往往致死或致慢性栓塞性肺动脉高压，相比而言PTE后果较前者更为严重^[5]^。

本研究通过对54例肺癌合并肺栓塞患者的临床资料进行回顾性分析，并以同期的162例不伴肺栓塞的肺癌患者作对照，以期探讨肺癌患者发生肺血栓栓塞的危险因素及对患者预后的影响。

## 1 材料与方法

### 1.1 研究对象及入选、排除标准

本研究对象均来自江苏省肿瘤医院和江苏省老年医院1999年4月－2010年1月收治的肺癌及肺癌合并肺栓塞患者。入组标准：肺癌诊断均为组织学或细胞学证实。PTE的诊断按中华医学会呼吸病学分会制定的《肺血栓栓塞症诊断和治疗指南》（草案）诊断标准^[6]^：①存在危险因素；②临床症状、体征。如不明原因的呼吸困难、胸痛、晕厥和休克；③结合心电图、X线胸片、动脉血气分析等基本检查，常规行D-二聚体检测（ELISA法，可作排除性诊断）；④螺旋CT肺动脉造影、核素肺V/Q显像、磁共振肺动脉造影或肺动脉造影等任一项检查阳性可确诊。排除标准：既往有血栓栓塞病史的患者。对每例PE患者选取同期入院3例肺癌作为对照，采用性别、年龄（±2岁）、治疗方式匹配。

### 1.2 资料采集

采集患者临床资料及实验室参数如下：性别、年龄、吸烟史、基础疾病、体力状况、既往治疗史；血细胞计数、血生化肝肾功能、肿瘤标记物、病理类型及分期、确诊PE影像学依据。

### 1.3 统计分析

应用STATA 11.0统计软件处理数据。连续性资料记录采用中位数和全距，分类资料采用百分数记录。危险因素筛选分析时：单因素分析，对于可能有意义的单变量（*P* < 0.20）进一步行条件*Logistic*回归分析。由于肺癌肺栓塞患者数量有限，我们构建两个回归模型。对两组人群的生存差异采用*Log*-*rank*检验。*P* < 0.05为有统计学差异。

## 2 结果

### 2.1 患者一般资料

本研究共包括54例肺栓塞患者（男/女: 35/19）和162例肺癌对照（男/女: 105/57）。肺栓塞组平均年龄为62岁（范围：39岁-86岁），对照组平均年龄为60岁（范围：37岁-86岁）。栓塞组腺癌占57.4%，小细胞肺癌占13%；早期肺癌占31.5%，中晚期肺癌占68.5%；48.2%的患者接受化疗治疗。92.6%的PTE患者（50/54）诊断依据影像学资料，另外4例患者依据临床表现、D-二聚体和血气分析而诊断。

### 2.2 单因素分析肺癌发生肺栓塞危险因素

纳入肺癌伴发肺栓塞的危险因素主要有：基础疾病、生活方式、治疗方案、病理类型、肿瘤分期、实验室指标。单因素分析结果显示两组间有差异的因素包括：慢性阻塞性肺病（chronic obstructive pulmonary disease, COPD）、化疗、中心静脉导管、腺癌、高血红蛋白、CEA升高。将*P* < 0.20的变量纳入多变量回归模型中进一步分析（表 1）。

**1:** 肺血栓栓塞危险因素单因素回归分析 Risk factors related to pulmonary thromboembolism of all patients by univariate analysis

	Cases	Controls	OR	95%CI	*P*
Comorbidities					
Cardiovascular disease	9	17	1.71	0.71-4.90	0.23
Diabetes	10	18	1.82	0.78-4.23	0.17
Hypertension	7	24	0.86	0.35-2.12	0.74
COPD	13	19	2.39	1.09-5.24	0.03
Life style					
Alcohol drinking	15	32	1.56	0.77-3.18	0.22
Smoking	15	29	1.76	0.86-3.62	0.12
Overweight	12	24	1.64	0.76-3.56	0.21
Type of management					
Surgery	13	41	0.94	0.46-1.92	0.86
Chemotherapy	26	41	2.74	1.44-5.20	< 0.01
Radiotherapy	11	29	1.17	0.54-2.55	0.67
Central venous catheters	27	54	2.00	1.07-3.74	0.03
Histological type					
Adenocarcinoma	31	52	2.85	1.52-5.37	< 0.01
Squamous cell carcinoma	14	39	1.10	0.54-2.24	0.78
Small-cell carcinoma	7	19	1.12	0.44-2.83	0.24
Stage					
Ⅰ-Ⅱ	17	39	1.45	0.74-2.85	0.28
Ⅲ-Ⅳ	37	69	2.93	1.53-5.64	< 0.01
Laboratory indicator					
Hemoglobin>140 g/L	27	45	2.60	1.38-4.90	< 0.01
Serum proteins < 60g/L	9	21	1.34	0.57-3.14	0.50
CEA>10 ng/mL	24	47	1.96	1.04-3.69	0.04
COPD: chronic obstructive pulmonary disease; OR: odds ratio.					

### 2.3 多变量回归分析

采用条件*Logistic*回归模型分析单因素分析有意义的变量。构建两个模型筛选相关变量，模型1：糖尿病，慢性阻塞性肺病，吸烟，化疗，中心静脉置管；模型2：腺癌，Ⅲ期-Ⅳ期，血红蛋白>140 g/L，CEA>10 ng/mL。结果显示接受化疗治疗患者发生栓塞风险增加了1.6倍。腺癌及晚期肿瘤患者风险也增加了1倍以上，此外还发现高血红蛋白血症也增加了栓塞风险（OR=2.10, *P*=0.03）。未发现糖尿病、慢性阻塞性肺病、吸烟、中心静脉导管及血CEA指标与肺栓塞风险有统计学意义（表 2）。

**2:** 肺癌肺血栓栓塞变量多因素分析结果 Multivariate analysis of the variables related to pulmonary thromboembolism in lung cancer

Parameter	OR	95%CI	*P*
Diabetes	1.23	0.81-2.76	0.46
COPD	2.39	0.79-3.21	0.12
Smoking	1.16	0.74-1.53	0.52
Chemotherapy	2.64	1.78-3.89	< 0.01
Central venous catheters	1.76	0.99-2.76	0.09
Adenocarcinoma	2.12	1.63-3.34	0.02
Stage Ⅲ-Ⅳ	2.25	1.72-3.29	0.05
Hemoglobin>140 g/L	2.10	1.44-3.08	0.03
CEA>10 ng/mL	1.37	0.76-2.66	0.63
Two models constructed: Model 1: Diabetes, COPD, Smoking, Chemotherapy, Central venous catheters; Model 2: Adenocarcinoma, stage Ⅲ-Ⅳ, Hemoglobin>140 g/L, CEA>10 ng/mL.			

### 2.4 肺癌伴/不伴肺栓塞患者的生存分析

肺癌合并肺栓塞患者中位生存时间为339天（95%CI: 180-667）；肺癌不伴肺栓塞患者中位生存时间为679天（95%CI: 462-798）。对两组患者采用*Log*-*rank*生存比较，肺栓塞组生存时间明显低于对照组（*P*=0.016）（图 1）。

**1:**
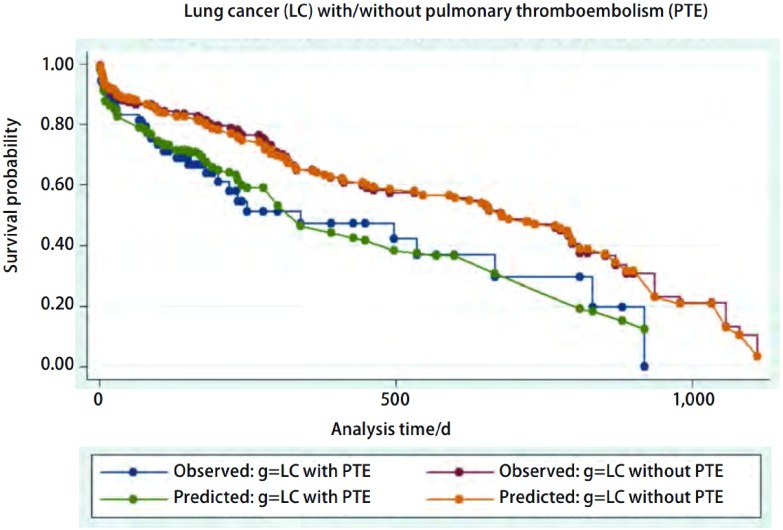
肺癌患者伴/不伴肺栓塞两组生存分析（*Kaplan*-*Meier*生存曲线及*Cox*预测曲线，*P*=0.016） Survival analysis between lung cancer patients with and without pulmonary thromboembolism (*Kaplan*-*Meier* and *Cox* predicting curves, *P*=0.016)

## 3 讨论

静脉血栓栓塞可发生于隐形癌或某种癌的并发症，也可以发生在住院、外科手术后以及其它疾病治疗过程中。其中危害最严重的是肺栓塞，占肿瘤患者住院期间主要死亡原因的第二位，尽管部分患者是由后来的尸体解剖证实^[7]^。此外一旦发生栓塞必定影响患者的抗肿瘤治疗，且抗凝治疗也可导致严重的出血事件。因此，了解肺癌患者肺栓塞危险因素、在诊治中加以警惕和预防显得尤为重要。

肺癌伴发肺栓塞者常由于机体血液的高凝状态所致。目前研究认为主要机制有：①肿瘤细胞产生组织因子Ⅲ，该组织因子与Ⅶ因子一起组成有效的促凝因子，进而通过外源性凝血途径直接激活因子Ⅹ；②肿瘤细胞可表达癌促凝素，它可以激活因子Ⅹ而不依赖其它凝血成分，导致血液中纤维蛋白、纤维蛋白原和纤维蛋白原降解产物升高而表现为血液高凝状态；③肿瘤细胞还可以通过淋巴细胞的介导激活单核细胞，间接激活凝血系统；④肿瘤细胞能分泌血管生长因子使微血管的通透性增加，使得肿瘤细胞所生成的凝血因子得以进入血管激活全身的凝血过程；⑤化疗、手术、深静脉置管等直接损伤血管内皮，触发凝血途径启动；⑥抗肿瘤支持治疗过程中使用促红细胞生成素、粒细胞集落刺激因子等也可引起引起血液的高凝状态等。

本研究发现接受全身性化疗治疗的肺癌者继发肺栓塞风险增高达1.6倍。这与Geerts等^[8]^报道的系统性全身化疗增加患者发生血栓的风险结论相一致。传统的化疗药物可能通过释放组织因子等激活体内凝血系统，然而新一代的分子靶向性药物中部分可引起血管内皮损伤直接激活血小板凝血途径。Zangari等^[9]^报道新的抗血管生成药物（包括贝伐珠单抗在内）可增加动静脉血栓形成风险，因此在选择靶向性药物治疗中应该予以关注。此前在胃癌、胰腺癌等腺癌中的观察发现栓塞发生率明显高于其它病理类型肿瘤，而本研究也发现肺腺癌增加肺栓塞发生风险。Geerts等^[8]^认为是腺癌分泌粘蛋白成分激活促凝因子导致血栓形成。晚期肿瘤发生栓塞所占比例高，预后差，已经得到了其它相关研究证实。肿瘤转移加重血液高凝状态，是其发生率增高的主要原因。在分析实验室指标预测栓塞风险时发现血红蛋白升高的患者发生栓塞比例高，可能与高血红蛋白致血液流变学改变，增加血液粘稠度，使血液淤滞，发生凝血异常有关。有报道^[10]^也发现血红蛋白升高与淋巴瘤发生静脉血栓形成相关。因此对于患者进行支持治疗时，需认识到促红细胞生成素可能在栓塞形成中的不利作用。

本组发生肺栓塞的患者有38例患者接受低分子肝素治疗，13例接受华法林治疗，另有3例患者因在发病一天内死亡而未能接受抗凝治疗。在研究肺癌发生肺栓塞患者与对照组患者进行生存分析时发现两者生存差异有统计学意义，肺栓塞组生存期明显短于对照组，与文献^[4]^报道结果一致。此外，有学者^[11]^发现在胰腺癌中肺栓塞也同样增加患者死亡率。因此，预防深静脉血栓形成是降低肿瘤相关肺栓塞死亡率的重要措施。

由于肿瘤合并肺栓塞具有高漏诊率、高病死率和病残率，一旦发生后果严重。因此，预防显得十分重要。最近，美国临床肿瘤协会、国家癌症综合协作网指南推荐，对住院恶性肿瘤患者、大手术治疗者、全身化疗同时接受反应停或来那度胺或地塞米松治疗的患者，建议行预防性抗凝治疗。本研究筛选出有意义的危险因素给临床治疗肺癌患者时预防肺栓塞提供参考，具有重要的临床意义。
